# Nano-MgO/AB decorated separator to suppress shuttle effect of lithium–sulfur battery†

**DOI:** 10.1039/c8na00420j

**Published:** 2019-02-12

**Authors:** Wenhao Sun, Xiaogang Sun, Qifan Peng, Hongyue Wang, Yunling Ge, Naseem Akhtar, Yaqin Huang, Kai Wang

**Affiliations:** Beijing Key Laboratory of Electrochemical Process and Technology for Materials, Beijing Laboratory of Biomedical Materials, Beijing University of Chemical Technology Beijing 100029 People’s Republic of China huangyq@mail.buct.edu.cn; Institute of Electrical Engineering, Chinese Academy of Sciences Beijing 100190 People’s Republic of China wangkai@mail.iee.ac.cn

## Abstract

Lithium–sulfur (Li–S) batteries are regarded as one of the most promising energy storage systems owing to their high specific energy, low cost and eco-friendliness. However, significant capacity fading caused by the shuttle of soluble polysulfides from the cathode to the anode significantly hampers their practical application. Here, we designed a nano-MgO/acetylene black (AB) decorated functional separator to suppress the shuttle of polysulfide intermediates, which can remarkably improve the electrochemical performance of Li–S batteries. Nano-MgO with the aid of the AB conductive network exhibits superior adsorption to polysulfides due to the synergistic effect of excellent chemisorption and improved electron conductivity. The electrochemical performance of the Li–S battery highly depends on the relative amount of nano-MgO and AB in the composite coating on the separator. A battery with the optimal decorated separator (MgO-25 separator, nano-MgO and acetylene black in the weight ratio 1 : 3) exhibits a high initial discharge capacity of 1238 mA h g^−1^ with high coulombic efficiency (∼97%) and retains a high capacity of 875 mA h g^−1^ after 100 cycles at 0.2 C. This study promotes the understanding of the synergistic effect of the polysulfide adsorbent and the conductive agent on the suppression of the shuttle effect, and provides a way to design polysulfide-blocking barriers for Li–S batteries.

## Abstract 2

A nano-MgO/AB decorated separator is developed to suppress the shuttle of polysulfides, achieving a synergistic effect of chemisorption and electronic conductivity.

## Graphical abstract

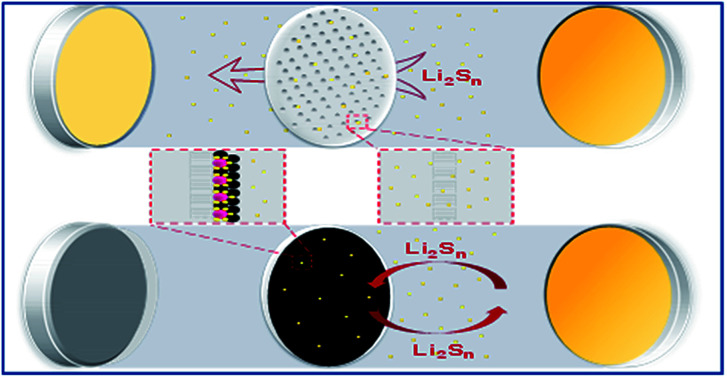

## Introduction

With the rapid development of electric vehicles and electronic mobile devices, energy storage systems with high energy density and long cycle life are highly required. However, the energy density of traditional lithium-ion batteries based on lithium intercalation electrochemistry is restricted by their low theoretical capacity.^1,2^ Rechargeable Li–S batteries composed of a sulfur cathode and a lithium–metal anode are considered as one of the most promising candidates for next-generation energy storage devices due to their large theoretical specific capacity of 1675 mA h g^−1^ and high theoretical energy density of 2600 W h kg^−1^. Additionally, sulfur as a cathode material is low-cost, naturally abundant and environmentally benign,^3–5^ which contributes to the commercialization of Li–S batteries. Nonetheless, the practical application of Li–S batteries is still hindered by several major issues: (i) the poor conductivity of sulfur reduces the utilization of the active material;^6^ (ii) the large volume change of the active material during the charge/discharge process can destroy the morphology and structure of the electrode and leads to the shedding of the active material;^7,8^ (iii) the shuttle phenomenon of dissolved polysulfide intermediates (Li_2_S_*n*_, 4 < *n* ≤ 8) from the cathode to the anode results in severe self-discharge, thus causing capacity loss and the short cycle life of the batteries.^9,10^

Over the years, extensive efforts have been devoted to addressing the “shuttle effect” in the Li–S battery system. One of the most popular methods is to trap sulfur and polysulfides within various hosts, including carbon (porous carbon,^11–15^ carbon nanotubes,^16,17^ graphene^18,19^ and elemental doped carbon^20,21^), conductive polymers,^22,23^ and metal–organic frameworks.^24,25^ Carbon materials are the most widely employed host, however, the trapped polysulfide still shows gradual loss following repeated cycles owing to the weak interaction of the physical adsorption and the open pore structure of the carbon material. Recently, researchers have proposed that a chemisorption interaction between sulfur and some hosts seems to be more efficient at trapping and immobilizing sulfur species. Compared to traditional carbon materials, metal oxides (such as TiO_2_,^26–29^ Al_2_O_3_,^30,31^ MnO_2_,^32^ Ti_4_O_7_,^33^ and Mg_0.6_Ni_0.4_O^34,35^) have excellent chemisorption ability by providing rich polar active sites for the anchoring of polysulfides. Among them, nano-MgO is considered as an especially good adsorbent for polysulfides. Previous work has also demonstrated that MgO as a cathode additive can improve the electrochemical performance of Li–S batteries.^36,37^ The hydrophilic nature of the nano-MgO nanoparticles and the presence of more electropositive Mg sites ensure that polysulfides can effectively be trapped on the cathode side of the separator. Moreover, Mg is more electropositive than the other metals, such as Ti and Mn, which promotes the increased electrostatic binding force between nano-MgO and the polysulfides.^36^

Recently, researchers have found that inserting a polysulfide barrier (so-called interlayer) between the cathode and the separator can adsorb polysulfide species.^38–42^ Directly coating a functional interlayer on the cathode side of the separator to block the shuttle of polysulfides is an effective strategy. Among various interlayer materials (carbon materials, metal oxides, and metal–organic frameworks), conductive carbon materials are particularly attractive, which can also act as expanded current collectors to reduce the cell resistance and to increase sulfur utilization. Similar to the sulfur cathode host, chemisorption can effectively suppress the shuttling of polysulfides. Due to the merits of nano-MgO, it is expected that the introduction of nano-MgO to the interlayer can effectively suppress polysulfide shuttling. However, it has rarely been reported to date.

**Scheme 1:**
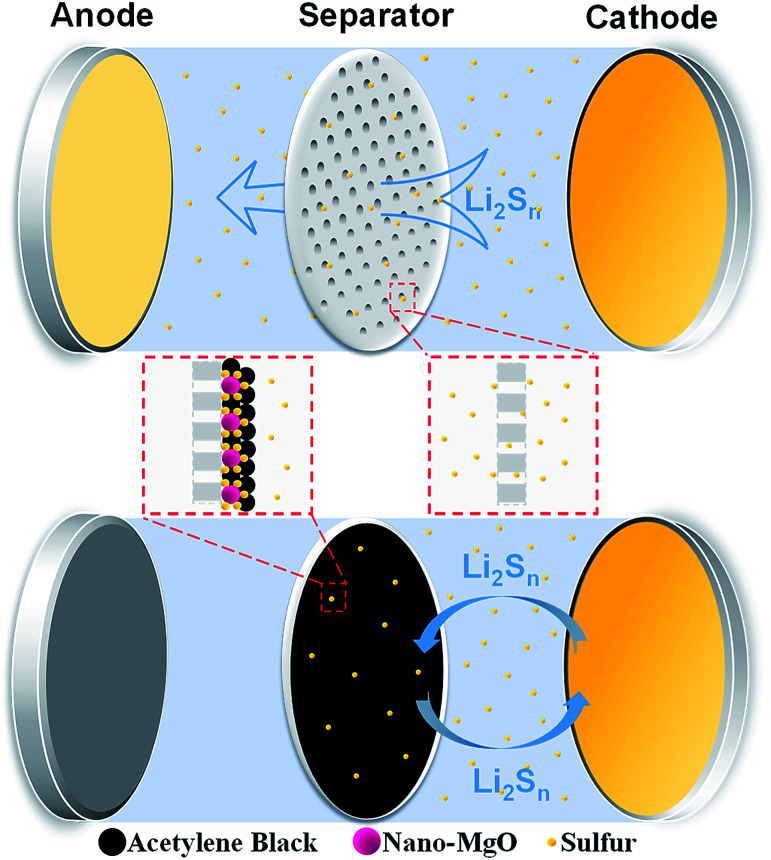
Illustration of the nano-MgO decorated separator inhibiting the diffusion of polysulfides.

Here, we present a nano-MgO/acetylene black (AB) decorated functional separator to inhibit the diffusion of polysulfides in Li–S batteries, as shown in Scheme 1. Nano-MgO with the aid of the AB conductive agent can achieve a synergistic effect of excellent chemisorption and superior electronic conductivity, which facilitates the adsorption and transformation reaction of polysulfides. Moreover, the battery performance highly depends on the relative amount of nano-MgO and AB in the composite interlayer of the separator. Insufficient nano-MgO cannot adsorb polysulfides sufficiently, resulting in poor cycling stability. On the other hand, excess nano-MgO results in an increase in the non-conductive area of battery, and polysulfides are anchored on the separator permanently without being reused, leading to low sulfur utilization. According to our experiment, a Li–S battery with the optimal separator (MgO-25 separator, MgO and AB in the weight ratio 1 : 3) exhibits better performance than other investigated batteries, and shows a high initial discharge capacity of 1238 mA h g^−1^ and a high capacity of 875 mA h g^−1^ after 100 cycles at 0.2 C.

## Experimental

### Preparation of the decorated separators

A 2 wt% gelatin solution was prepared by adding an appropriate amount of gelatin into deionized water. Five slurries with different weight ratios of nano-MgO and acetylene black (AB) were prepared. Nano-MgO and acetylene black (AB) were firstly mixed in various ratios by ball-milling for 5 h at 25 °C. The content of gelatin in all slurries is the same. The weight ratios of nano-MgO and AB in the first three decorated separators are 1 : 3, 3 : 3 and 9 : 3, which are defined as the MgO-25 separator, the MgO-50 separator and the MgO-75 separator, respectively. The MgO-100 separator and the AB separator consist only of nano-MgO and AB, respectively. The slurries were then coated on Celgard 2400 separators with an applicator, and were subsequently dried in a vacuum chamber at 60 °C for 24 h. Lastly, the decorated separators were tablet-pressed and punched into circles with a diameter of 19 mm. The loading mass of the decorated separators was controlled between 0.2–0.4 mg.

### Fabrication of the cathode

The slurry of the common sulfur cathode was prepared by mixing sublimed sulfur, AB and gelatin (2 wt% gelatin solution) in the weight ratio 63 : 30 : 7, respectively. The slurry of the pristine MgO cathode was prepared by mixing nano-MgO, AB and gelatin in the weight ratio 20 : 60 : 20, respectively. After ball-milling for 5 h at 25 °C, the slurries were evenly coated on aluminum foil with an applicator and dried in a vacuum chamber at 60 °C for 24 h. Finally, the cathodes were tablet-pressed and punched into circles with a diameter of 12 mm. The loading mass of sulfur in the sulfur cathode is 1.0–1.5 mg.

### Preparation of the Li_2_S_6_-containing solution

In a glove box under an argon atmosphere, the sublimed sulfur and Li_2_S with a molar ratio of 5 : 1 were mixed in tetrahydrofuran solvent. After 24 hours of magnetic stirring, the Li_2_S_6_-containing solution was prepared.

### Materials characterization

The surface morphologies of the differently decorated separators and the lithium–metal after cycling were characterized by scanning electron microscopy (SEM, HITACHIS-4800) and high resolution transmission electron microscopy (HRTEM, HITACHIH-800). The elemental mapping check was performed using an energy dispersive spectrometer. The sulfur content of the differently decorated separator coatings was determined by thermogravimetric analysis (TGA, Hengjiu, China) in the temperature range 25–800 °C under an air atmosphere, with a heating rate of 10 °C min^−1^ and an air-flow rate of 100 mL min^−1^. The crystalline phases of the nano-MgO particles and the MgO-25 separator slurry were observed by X-ray diffraction (XRD, D/max-2500/PC, Rigaku) from 10° to 90° with Cu Kα radiation (*λ* = 0.154 nm). UV-visual spectroscopy (UV/vis, SEC2000-DH) was used to analyze the adsorption effect of nano-MgO and AB on lithium polysulfide. The conductivity and wettability of the decorated separators were measured by a four-probe meter and a contact angle meter, respectively.

### Electrochemical measurements

Coin-type (CR2025) cells were assembled and sealed in an argon-filled glove box, where the oxygen and water contents were less than 1 ppm. Celgard 2400 separators with/without decorated interlayers were used as the separator, and lithium–metal was used as the anode. 30 μL of electrolyte was used, which consisted of 1 mol L^−1^ lithium bis(trifluoromethanesulfonyl)imide (LiTFSI) and a solvent of 1,3-dioxolane (DOL) and 1,2-dimethoxyethane (DME) (1 : 1, in volume) with 0.4 mol L^−1^ LiNO_3_ additive. The galvanostatic charge–discharge cycling of the batteries was performed in a potential range of 1.7–2.8 V (*vs.* Li/Li^+^) by a LAND-CT2001A instrument. Electrochemical impedance spectroscopy (EIS) was performed on a Solartron 1280 Z at an open-circuit voltage ranging from 100 MHz to 10 kHz at room temperature. Cyclic voltammetry (CV) tests were carried out with a Solartron 1280Z at a scan rate of 0.1 mV s^−1^.

## Results and discussion

**Fig. 1:**
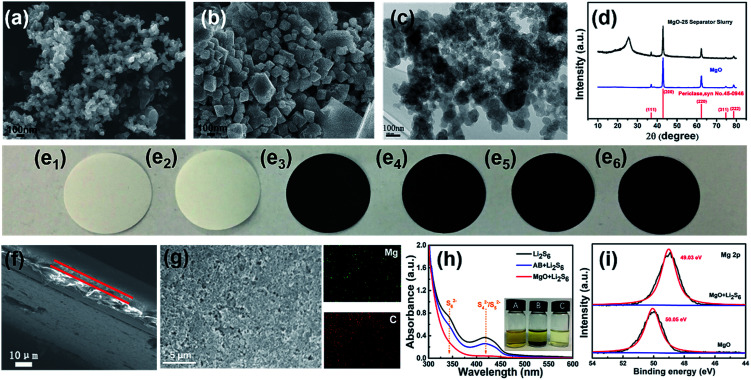
SEM images of the (a) AB and (b) nano-MgO particles. (c) HRTEM image of the MgO-25 separator slurry. (d) XRD patterns of the nano-MgO particles and the MgO-25 separator slurry. Digital images of (e_1_) the Celgard 2400 separator, (e_2_) the MgO-100 separator, (e_3_) the MgO-75 separator, (e_4_) the MgO-50 separator, (e_5_) the MgO-25 separator and (e_6_) the AB-coated separator. (f) Cross-sectional SEM image of the MgO-25 separator. (g) SEM image and EDS spectra of the MgO-25 separator. (h) Digital image and UV-vis spectroscopy of the pristine and mixed Li_2_S_6_ solution with nano-MgO and AB powder. (Inset (A) pristine Li_2_S_6_ solution, inset (B) AB + Li_2_S_6_ solution, inset (C) nano-MgO + Li_2_S_6_ solution.) (i) XPS spectra of the Mg 2p region of nano-MgO before and after adsorbing Li_2_S_6_ solution.

From the SEM images of AB (Fig. 1a) and nano-MgO (Fig. 1b), the main particle size of AB and nano-MgO is ∼50 nm and ∼100 nm, respectively. The employed nano-MgO has obvious lattice fringes and the lattice spacing is estimated to be 0.2125 nm as shown in Fig. S1,† which is consistent with that of standard MgO (JCPDS no. 45-0946, *d* = 0.21056 nm). Furthermore, the HRTEM image of the MgO-25 separator slurry is shown in Fig. 1c. The distribution of the nano-MgO particles in the slurry looks relatively uniform, demonstrating the sufficient blending of AB and nano-MgO in the composite interlayer. According to Fig. 1d, the nano-MgO and MgO-25 separator slurries both show characteristic peaks of MgO, including (111), (200), (220), (311), and (222) in the XRD patterns. The diffraction peak of the MgO-25 separator slurry at 2*θ* = 26° and is attributed to the existence of AB. The results indicate that nano-MgO can maintain the original crystal shape well when it has been prepared as decorated separator slurries. Fig. 1e shows photographs of the six separators involved in this study, which are (e_1_) the Celgard 2400 separator, (e_2_) the pristine MgO-coated (MgO-100) separator, (e_3_) the MgO-75 separator, (e_4_) the MgO-50 separator, (e_5_) the MgO-25 separator and (e_6_) the pristine AB-coated separator, respectively. The interlayer coating looks quite smooth, confirming that the functional slurries can uniformly coat on the Celgard 2400 separators. Fig. 1f and S2† are cross-section SEM images of the separators, which reveal that the thickness of the commercial Celgard 2400 separator is about 20 μm. The nano-MgO/AB interlayer is around 5 μm for all of the decorated separators except the MgO-100 separator, for which the interlayer thickness is around 10 μm. Fig. 1g, S3 and S4† are SEM images and EDS spectra of the MgO-25 separator, the MgO-50 separator and the MgO-75 separator, respectively, which further prove that nano-MgO is uniformly distributed in the composite interlayer of the separators. Moreover, the results indicate that the AB conductive network is established in the interlayer of the separators, which facilitates ion/electron transport and accelerates the transformation of the trapped polysulfides during electrochemical cycling.

Prior to making the Li–S battery with the nano-MgO decorated separator, we investigated the interaction between polysulfides and nano-MgO by directly adding nano-MgO powder into a Li_2_S_6_ solution. As shown in Fig. 1h, the solution became clear immediately, showing that nano-MgO can adsorb polysulfides effectively. As a control experiment, the adsorption effect of pristine AB to polysulfides is very weak, which was further confirmed by UV-visual spectroscopy. The peak located at 330–350 nm (ref. 43 and 44) is ascribed to the existence of Li_2_S_6_ and becomes weak when adding nano-MgO, showing the strong chemisorption interaction between nano-MgO and Li_2_S_6_. To further confirm the interaction between polysulfides and nano-MgO, we collected XPS spectra of the Mg 2p region of nano-MgO before and after adsorbing Li_2_S_6_ solution as shown in Fig. 1i. From the XPS spectra, the Mg 2p spectrum of MgO/Li_2_S_6_ shows a 1.02 eV shift to a lower binding energy, indicating that there is electron transfer from Li_2_S_6_ to Mg ions. The wettability of the separator can affect the infiltration of the electrolyte and the diffusion rate of ions during the charging and discharging process. Contact angle tests were carried out to evaluate the electrolyte wettability properties on the separator. As the organic electrolyte of Li–S batteries is seriously volatile, the contact angles of the six different separators were measured with deionized water, which possesses similar polarity to the electrolyte of Li–S batteries. From Fig. S5,† the water contact angle on the pristine Celgard 2400 separator is close to 90.0°. However, the water contact angles on the decorated separators gradually decrease with the increase in nano-MgO content, indicating that the wettability of the separators improves with the nano-MgO functional coating.

**Fig. 2:**
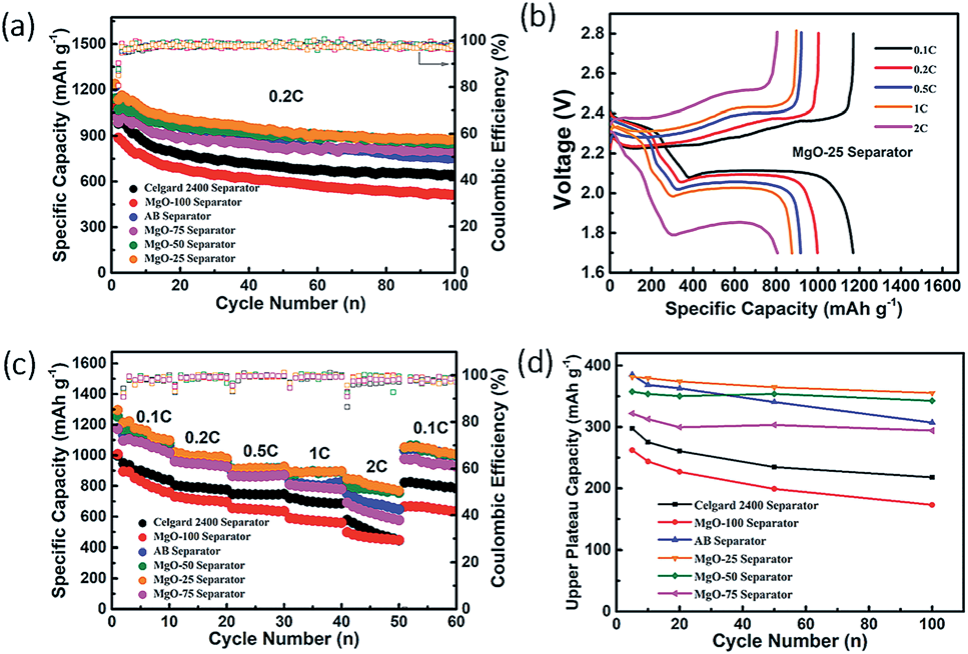
(a) Cycling performance of the batteries with various separators at 0.2 C; (b) charge/discharge voltage curves at different rates for different batteries with the MgO-25 separator; (c) rate capability of the batteries with the various separators; (d) first discharge plateau capacities of the different batteries at a rate of 0.2 C.

To study the electrochemical performance of the Li–S batteries with the MgO coating, the pristine MgO cathode (without sulfur) and the Li anode were first assembled into a Li–MgO battery. As shown in Fig. S6,† there are no apparent redox peaks appearing in the cyclic voltammetry (CV) curves of the Li–MgO battery in the working voltage range 1.7 V to 2.8 V. Furthermore, Fig. S6b† shows that the discharge specific capacity of the Li–MgO battery is only 1.6 mA h g^−1^ and there is no change for the 100 charge–discharge cycles, indicating that nano-MgO has no extra capacity contribution to the Li–S battery in its operating voltage range. Fig. 2a and Table S1† show the capacity and cycling performance of the Li–S batteries with the various separators. Among the Li–S batteries with the various separators, the battery with the MgO-25 separator has the largest initial discharge specific capacity of 1238 mA h g^−1^ with a high coulombic efficiency (∼97%), and retains a specific capacity of 875 mA h g^−1^ after 100 charge–discharge cycles at 0.2 C. The initial discharge specific capacity of the Li–S battery with the AB decorated separator is also very high, however, the capacity retention rate is only 61.82% after 100 cycles. This result demonstrates that nano-MgO exhibits a synergistic effect and improves both the capacity and the cycling performance of battery considerably. The charge–discharge curves of the Li–S batteries at various rates are shown in Fig. 2b and S7.† All curves display the typical characteristics of Li–S batteries with two discharge plateaus and one charge plateau, corresponding to the electrochemical redox reaction of the Li–S battery. The first discharge plateau (FDP) at about 2.35 V is attributed to the transformation of elemental sulfur into soluble polysulfides (Li_2_S_*n*_, 4 ≤ *n* ≤ 8) and the following discharge plateau at about 2.05 V is attributed to the transformation of the long-chain polysulfides into insoluble polysulfides (Li_2_S_2_ or Li_2_S). The charge plateaus are ascribed to the transformation of Li_2_S_2_/Li_2_S into S_8_. With increasing rate, the voltage gap between the discharge and charge plateaus is extended, caused by sulfur that has not reacted at high rates. The plateaus are flatter and the polarization is smaller for the Li–S battery with the MgO-25 separator than that of the batteries with other separators, suggesting a kinetically efficient reaction process. From Fig. 2c, the discharge specific capacity of the Li–S battery with the MgO-25 separator is 1252.2 mA h g^−1^ at 0.1 C, and is still 805.3 mA h g^−1^ at a rate of 2 C, exhibiting superior rate capability of all the batteries with decorated separators. Furthermore, it can recover 1121.9 mA h g^−1^ with a high capacity retention ratio of 95.9% when the discharge rate returns to 0.1 C. The ultrahigh reversibility demonstrates that the MgO/AB composite coating on the MgO-25 separator can effectively react with the trapped polysulfides during the charge–discharge process. The first discharge plateau (FDP) analysis is a pre-assessment for polysulfide diffusion and the well-retained FDP capacitance manifests good reversible electrochemical performance, which also demonstrates the effective inhibition of polysulfide diffusion as the FDP relates to the formation of highly soluble polysulfides.^45^ The first discharge plateau (FDP) capacity of various batteries has been studied at a rate of 0.2 C, as shown in Fig. 2d. When employing the MgO-25 separator, the FDP capacity is 380.2 mA h g^−1^, which is approximately 90.7% of the theoretical capacity (419 mA h g^−1^). Moreover, the FDP capacity of the Li–S battery with the MgO-25 separator retains 84.82% of the theoretical specific capacity after 100 cycles, while the Li–S batteries with the MgO-50 separator, the AB separator, the MgO-75 separator, the Celgard 2400 separator and the MgO-100 separator show a capacity retention of 81.8%, 73.5%, 70.1%, 52.0% and 40.9%, respectively. Although the initial FDP capacity of the battery with the AB separator is 386.5 mA h g^−1^ (nearly 92.2% of the theoretical value), the capacity shows continuous decay due to the serious “shuttle effect”. The above results demonstrate that the MgO-25 separator can trap polysulfides and react with the trapped species effectively due to the synergistic effect of nano-MgO and AB.

**Fig. 3:**
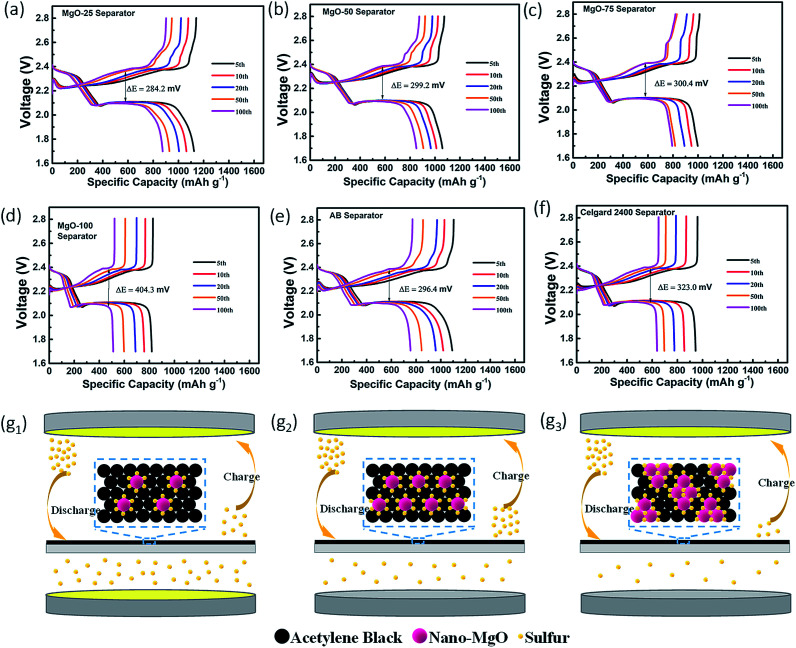
Charge/discharge profiles at 0.2 C of the Li–S batteries with (a) the MgO-25 separator, (b) the MgO-50 separator, (c) the MgO-75 separator, (d) the MgO-100 separator, (e) the AB separator and (f) the Celgard 2400 separator, respectively. Illustrations of the polysulfides-trapped/reactivated mechanisms of the differently decorated separators with (g_1_) insufficient, (g_2_) moderate and (g_3_) excess nano-MgO.

The charge/discharge profiles of the Li–S batteries with the various separators at a rate of 0.2 C are presented in Fig. 3a–f. Among them, the profile of the Li–S battery with the MgO-25 separator exhibits the smallest potential difference (Δ*E*) between the discharge and charge plateaus due to the synergistic effect of strong chemisorption and the superior conducting network. However, the pristine Celgard separator and the pure MgO decorated separator show large Δ*E* values because their poor conductivity limits the secondary reaction of the adsorbed polysulfides. The pure AB coating cannot adsorb polysulfides effectively which also causes poor charge–discharge reversibility. The relative amount of MgO and AB influences the performance of the Li–S batteries greatly. As illustrated in Fig. 3g_1_–g_3_, the insufficient nano-MgO cannot adsorb polysulfides sufficiently, which is proposed to result in poor cycling stability. However, excess nano-MgO (such as the MgO-50 and MgO-75 separators) causes an increase in the non-conductive area of the battery, and polysulfides will anchor on the separator permanently without being reused, leading to low sulfur utilization. In this case, excess MgO restricts the reaction kinetics and causes poor charge–discharge reversibility.

**Fig. 4:**
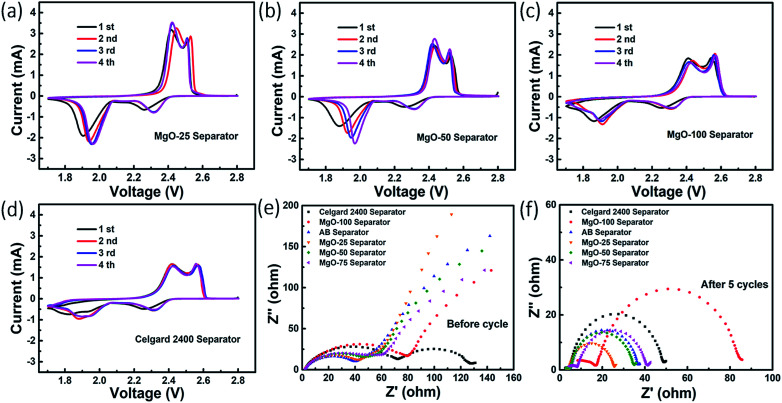
CV curves of the batteries with (a) the MgO-25 separator, (b) the MgO-50 separator, (c) the MgO-100 separator and (d) the Celgard 2400 separator. EIS spectra of the batteries with various separators (e) before cycling and (f) after 5 cycles.

To further understand the synergistic effect of nano-MgO and AB on the reaction kinetic process of Li–S batteries, the CV and EIS of the batteries were investigated. Fig. 4a–d and S8† show the first four CV cycles of the Li–S batteries with the various separators at 0.1 mV s^−1^. The reduction peaks at ∼2.35 V and ∼1.94 V are assigned to the transformation of sulfur to soluble polysulfides (Li_2_S_*n*_, 4 ≤ *n* ≤ 8) and the long-chain polysulfides to insoluble Li_2_S_2_/Li_2_S, respectively. In the subsequent anodic scan, the main peak at ∼2.44 V relates to the transformation of Li_2_S to high-order polysulfides and sulfur. For all the assembled Li–S batteries, the reduction peak shifts right gradually with increasing CV cycles, caused by irreversible diffusion of sulfur into the electrolyte and the corresponding electrode polarization. The CV curves of the Li–S battery with MgO-25 separator gradually stabilise as the CV cycles increase due to uniform secondary distribution of the sulfur on the cathode surface, suggesting excellent cycling stability and reversibility. In the fourth cycle, the voltage gaps (Δ*E*) between the oxidation and reduction peaks of the Li–S batteries with the AB separator, the MgO-25 separator, the MgO-50 separator, the MgO-75 separator, the MgO-100 separator and the Celgard 2400 separator are 589.6 mV, 544.43 mV, 552.98 mV, 638.12 mV, 674.13 mV, and 662.23 mV, respectively. This indicates that the battery with the MgO-25 separator possesses the smallest Δ*E*. In contrast to the other Li–S batteries, the CV curves of the battery with the MgO-25 separator exhibit sharper peaks, demonstrating less polarization. Thus, only the appropriate amount of MgO and AB exhibits the synergistic effect of excellent chemisorption and superior electron conductivity.

Fig. 4e shows Nyquist plots of the Li–S batteries with various decorated separators. The semicircle in the high-frequency region represents the charge transfer resistance (*R*_ct_) at the interface between the cathode and the electrolyte, while the inclined line in the low-frequency region corresponds to the Warburg impedance produced from the diffusion of electrolyte ions in the electrode. The Nyquist plots of the Li–S batteries with the AB separator and the MgO-25 separator almost overlap in the high frequency region and show the smallest *R*_ct_, which confirms their excellent electronic conductivity. However, a second semicircle appears in the intermediate-frequency region for the battery with the pristine Celgard 2400 separator, which is attributed to battery self-discharge during the static process, indicating increased resistance. For all the tested batteries, another semicircle appears in the medium-to-high frequency region after 5 cycles at a rate of 0.5 C, representing the deposition resistance (*R*_d_) of Li_2_S/Li_2_S_2_ (ref. 46) as shown in Fig. 4f. It is worth noting that the *R*_ct_ and *R*_d_ values of the battery with the MgO-25 separator are the smallest among all of the as-prepared batteries that are presented in detail in Table S2.† The significant reduction in resistance further confirms the synergistic effect of nano-MgO and AB, where the AB conductive network facilitates electron conduction during the reactivation of the adsorbed polysulfides.

**Fig. 5:**
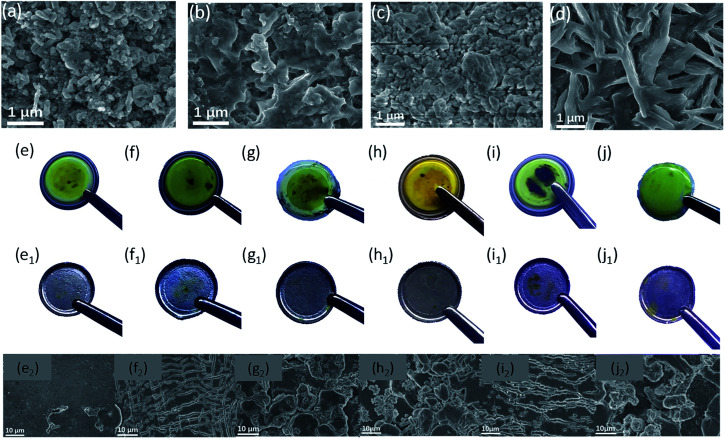
Surface morphology of the MgO-25 separator and the MgO-100 separator (a and c) before and (b and d) after cycling, respectively. Photographs of the various separators after 5 cycles: (e) MgO-25 separator, (f) MgO-50 separator, (g) MgO-75 separator, (h) MgO-100 separator (i) AB separator, and (j) Celgard 2400 separator. Photographs and corresponding SEM images of the Li–metal anodes of the batteries with various separators: (e_1_ and e_2_) MgO-25 separator, (f_1_ and f_2_) MgO-50 separator, (g_1_ and g_2_) MgO-75 separator, (h_1_ and h_2_) MgO-100 separator, (i_1_ and i_2_) AB separator, and (j_1_ and j_2_) Celgard 2400 separator.

*Ex situ* observation of the separator and the Li–metal anode before and after charge–discharge cycling was performed to validate the polysulfide adsorption effect. The surface morphology of the MgO-25 separator (Fig. 5a and b) and the MgO-100 separator (Fig. 5c and d) before and after cycling were checked by SEM. The nano-MgO particles uniformly distribute on the surface of the separators before cycling. After 100 cycles at a rate of 0.2 C, polysulfides deposit on the surface of the MgO-25 separator due to the strong interaction between MgO and the polysulfides. In contrast, a thick polysulfide aggregation layer on the surface of the MgO-100 separator is observed after cycling, which is due to excess nano-MgO adsorbing many more polysulfides which cannot transform into sulfur sufficiently without the aid of the AB conducting network. Fig. 5e–j show photographs of the various separators after 5 charge–discharge cycles at a rate of 0.5 C. The surface of the separators with nano-MgO in Fig. 5e–h are dark orange, implying more polysulfides are adsorbed on the surface. However, the color of the AB decorated separator and the pristine Celgard 2400 separator appears light orange, demonstrating a weak adsorption to polysulfides (Fig. 5i and j). Surface photographs (Fig. 5e_1_–j_1_) and SEM images (Fig. 5e_2_–j_2_) of the Li–metal anode of the batteries are also shown. From Fig. 5e_1_ and e_2_, the surface of the lithium anode in the battery with the MgO-25 separator is the smoothest, meaning that the MgO-25 separator can effectively prevent polysulfides from depositing on the Li–metal anode. Thus, the *ex situ* characterization results further confirm that the MgO-25 decorated separator can effectively adsorb polysulfides and suppress the shuttle effect of Li–S batteries.

## Conclusions

In summary, a nano-MgO/AB decorated separator is developed to suppress the shuttle of polysulfide intermediates for the first time. Nano-MgO with the aid of an AB conductive agent can achieve a synergistic effect of excellent chemisorption and superior electronic conductivity, which can adsorb polysulfides effectively and accelerate the reaction kinetics for the transformation of polysulfides into sulfur. Moreover, it is found that the battery performance highly depends on the relative amount of nano-MgO and AB in the composite interlayer of the separator. Insufficient nano-MgO cannot adsorb polysulfides sufficiently, resulting in poor cycling stability. On the other hand, excess nano-MgO results in an increase in the non-conductive area of the battery, and polysulfides would be anchored on the separator permanently without being reused, leading to low sulfur utilization. According to our experiments, the Li–S battery with the optimal separator (MgO-25 separator, MgO and AB in the weight ratio 1 : 3) exhibits a high initial discharge capacity of 1238 mA h g^−1^ with high coulombic efficiency (97%) and retains a high capacity of 875 mA h g^−1^ after 100 cycles at 0.2 C. This study promotes the understanding of the synergistic effect of the polysulfide chemical adsorbent and the conductive agent on the suppression of the “shuttle effect” of Li–S batteries, which is also helpful to guide the design of high-performance functional separators for practical high-energy-density Li–S batteries.

## Conflicts of interest

There are no conflicts to declare.

## Supplementary Material

NA-001-C8NA00420J-s001
