# Supplementary material for: Nano-MgO/AB decorated separator to suppress shuttle effect of lithium–sulfur battery
Source: Nanoscale Adv. 2019 Feb 12;1(4):1589–97. doi: 10.1039/c8na00420j (PMC9419172; doi:10.1039/c8na00420j)
Supplement: NA-001-C8NA00420J-s001 [file NA-001-C8NA00420J-s001.pdf]

Electronic Supplementary Material (ESI) for Nanoscale Advances.
This journal is © The Royal Society of Chemistry 2019

Electronic supplementary information

Nano-MgO/AB Decorated Separator to Suppress Shuttle Effect of Lithium-Sulfur Battery

Wenhao Sun[1,&], Xiaogang Sun[1,&], Qifan Peng[1], Hongyue Wang, [1,2] Yunling Ge[1], Naseem Akhtar[1], Yaqin Huang[1,*], Kai Wang[2,*]

[1]Beijing Key Laboratory of Electrochemical Process and Technology for Materials, Beijing Laboratory of Biomedical Materials, Beijing University of Chemical Technology, Beijing 100029, People's Republic of China.

[2]Institute of Electrical Engineering, Chinese Academy of Sciences, Beijing 100190, People's Republic of China.

*E-mail: huangyq@mail.buct.edu.cn; wangkai@mail.iee.ac.cn

&These authors contributed equally to this work and should be considered co-first authors.

Table S1 The discharge specific capacity of the Li-S batteries with various separators at 0.2C.

Modified separator	Initial discharge specific capacity (mAh g^{-1})	100th discharge specific capacity (mAh g^{-1})
AB Separator	1220.8	754.7
MgO-25 Separator	1238.1	875
MgO-50 Separator	1141.9	850.5
MgO-75 Separator	1033.1	793.9
MgO-100 Separator	1080	511.6
Celgard 2400 separator	1059.5	638.7

Table S2 Electrical conductivity of the various as-prepared separator.

Modified separator	25℃ electrical resistivity (Ω/m)	25℃ electrical conductivity (S/m)
AB Separator	293.717	3.405×10^{-3}
MgO-25 Separator	623.189	1.605×10^{-3}
MgO-50 Separator	1005.079	0.995×10^{-3}
MgO-75 Separator	8074.907	0.124×10^{-3}

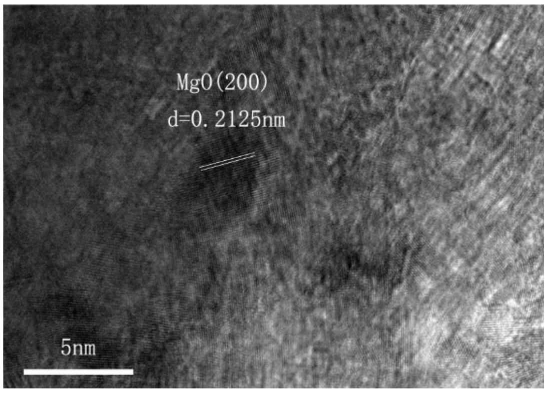

Fig.S1. The HRTEM images of nano-MgO.

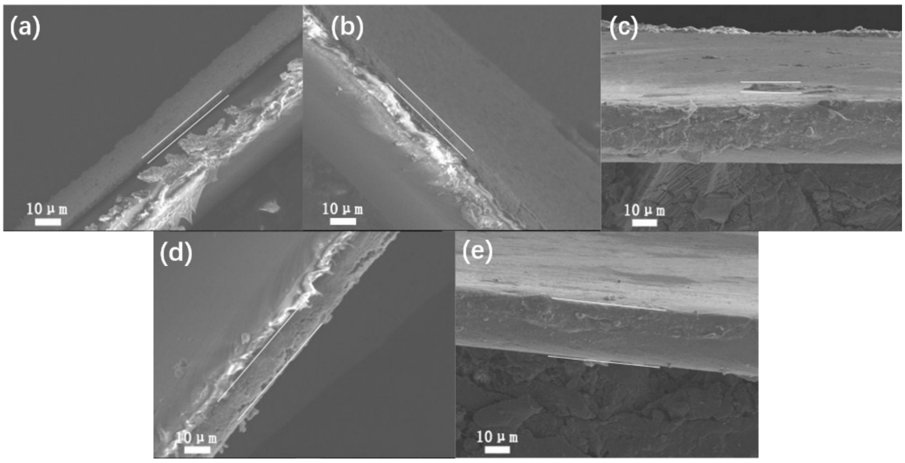

Fig.S2. The cross-sectional images of (a) MgO-50 separator, (b) MgO-75 separator, (c) MgO-100 separator, (d) AB separator and (e) Celgard 2400 separator.

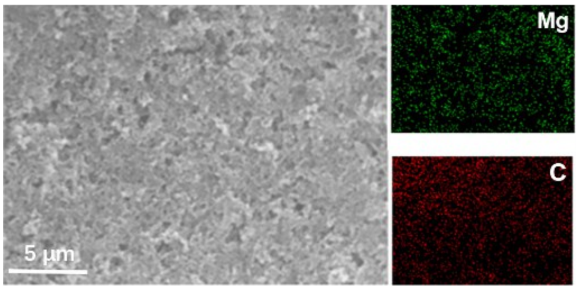

Fig.S3. The SEM images and the EDS spectrums of MgO-50 separator.

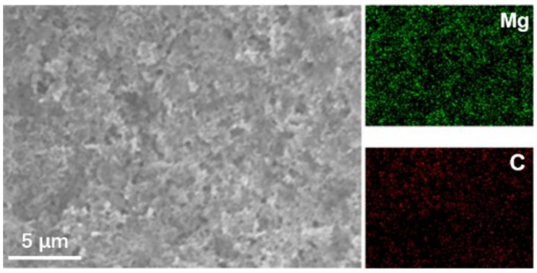

Fig.S4. The SEM images and the EDS spectrums of MgO-75 separator.

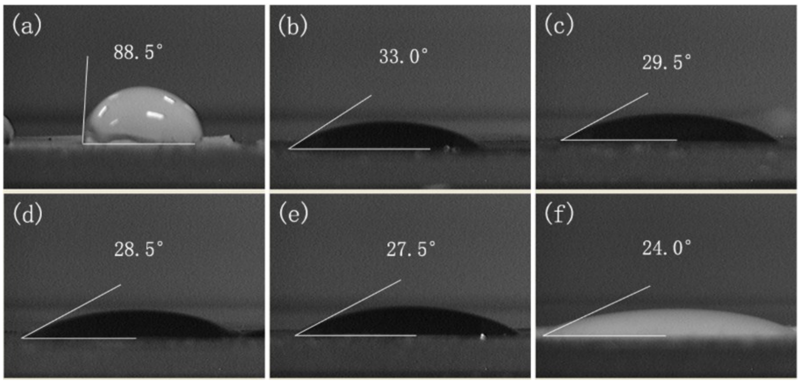

Fig.S5. The water contact angle for (a) Celgard 2400 separator, (b) AB separator, (c) MgO-25 separator, (d) MgO-50 separator, (e) MgO-75 separator and (f) MgO-100 separator.

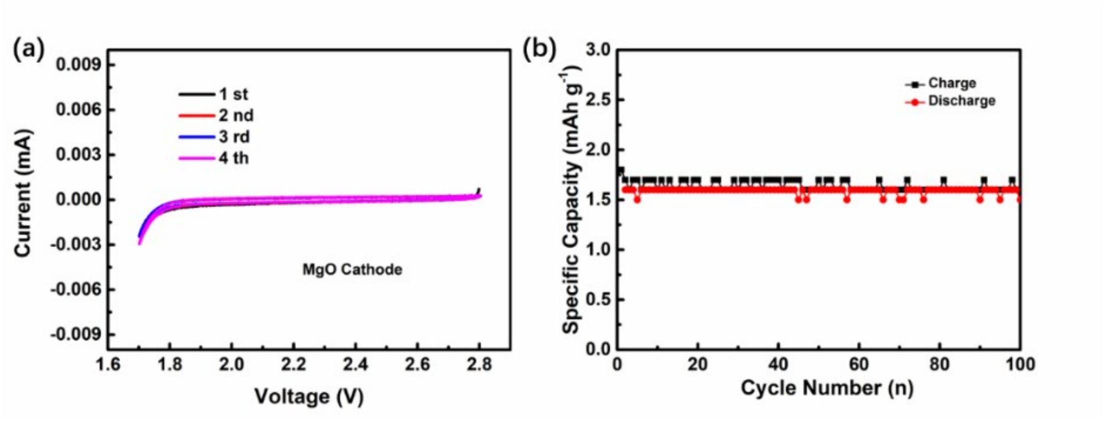

Fig.S6. (a) The CV curve and (b) cycling performance at the current of 100mA g^{-1} of the lithium-MgO battery.

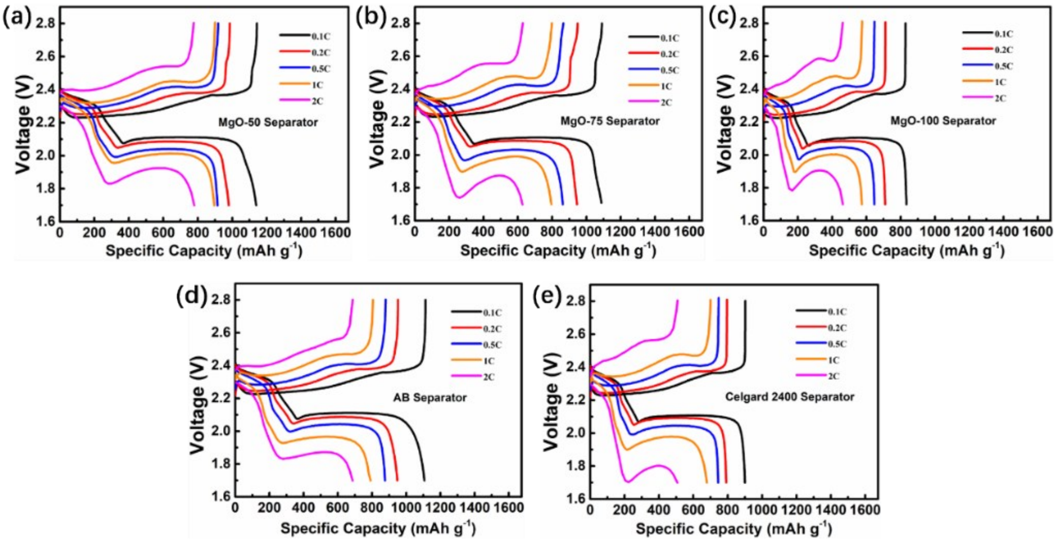

Fig.S7. The charge/discharge voltage curves at different rates for different batteries with (a) MgO-50 separator, (b) MgO-75 separator, (c) MgO-100 separator, (d) AB separator and (e) Celgard 2400 separator.

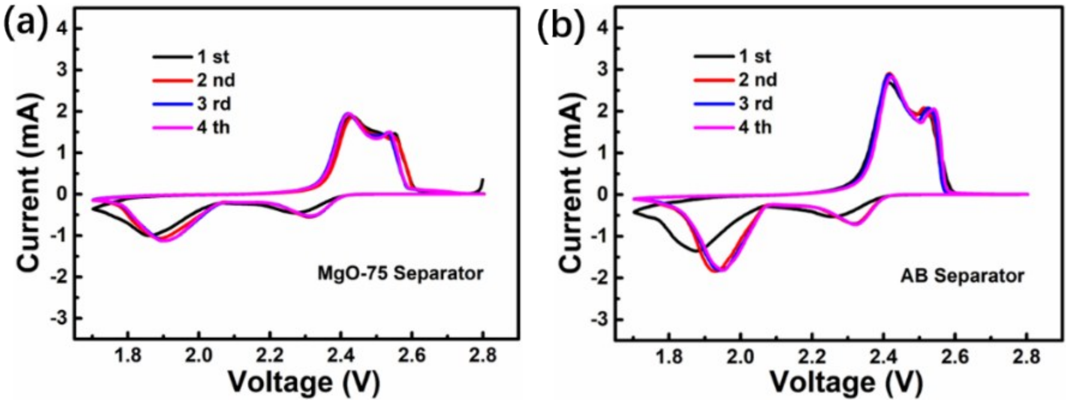

Figure.S8. The CV curves of batteries with (a) MgO-75 separator, (b) AB separator.
